# Budget impact analysis of using procalcitonin to optimize antimicrobial treatment for patients with suspected sepsis in the intensive care unit and hospitalized lower respiratory tract infections in Argentina

**DOI:** 10.1371/journal.pone.0250711

**Published:** 2021-04-30

**Authors:** Osvaldo Ulises Garay, Gonzalo Guiñazú, Wanda Cornistein, Javier Farina, Ricardo Valentini, Gabriel Levy Hara

**Affiliations:** 1 Market Access and Medical Affairs, Roche Diagnostics, Buenos Aires, Argentina; 2 Ricardo Gutiérrez Children’s Hospital, Buenos Aires, Argentina; 3 Infection Control, Hospital Austral, Buenos Aires, Argentina; 4 Hospital Cuenca Alta Néstor Kirchner, Buenos Aires, Argentina; 5 CEMIC University Hospital, Buenos Aires, Argentina; 6 Unit of Infectious Diseases, Hospital Carlos G Durand, Buenos Aires, Argentina; National Center for Global Health and Medicine, JAPAN

## Abstract

**Background:**

Inappropriate antibiotic use represents a major global threat. Sepsis and bacterial lower respiratory tract infections (LRTIs) have been linked to antimicrobial resistance, carrying important consequences for patients and health systems. Procalcitonin-guided algorithms may represent helpful tools to reduce antibiotic overuse but the financial burden is unclear. The aim of this study was to estimate the healthcare and budget impact in Argentina of using procalcitonin-guided algorithms to guide antibiotic prescription.

**Methods:**

A decision tree was used to model health and cost outcomes for the Argentinean health system, over a one-year duration. Patients with suspected sepsis in the intensive care unit and hospitalized patients with LRTI were included. Model parameters were obtained from a focused, non-systematic, local and international bibliographic search, and validated by a panel of local experts. Deterministic and probabilistic sensitivity analyses were performed to analyze the uncertainty of parameters.

**Results:**

The model predicted that using procalcitonin-guided algorithms would result in 734.5 [95% confidence interval (CI): 1,105.2;438.8] thousand fewer antibiotic treatment days, 7.9 [95% CI: 18.5;8.5] thousand antibiotic-resistant cases avoided, and 5.1 [95% CI: 6.7;4.2] thousand fewer *Clostridioides difficile* cases. In total, this would save $422.4 US dollars (USD) [95% CI: $935;$267] per patient per year, meaning cost savings of $83.0 [95% CI: $183.6;$57.7] million USD for the entire health system and $0.4 [95% CI: $0.9;$0.3] million USD for a healthcare provider with 1,000 cases per year of sepsis and LRTI patients. The sensitivity analysis showed that the probability of cost-saving for the sepsis patient group was lower than for the LRTI patient group (85% vs. 100%).

**Conclusions:**

Healthcare and financial benefits can be obtained by implementing procalcitonin-guided algorithms in Argentina. Although we found results to be robust on an aggregate level, some caution must be used when focusing only on sepsis patients in the intensive care unit.

## Background

Antibiotics are extensively used for a broad range of bacterial infections and are essential in the treatment of patients with sepsis and bacterial respiratory tract infections. The World Health Organization warns that inappropriate antibiotic use represents a major global health threat [1]. Studies have shown that up to 50% of antibiotic prescriptions in hospitals are either inappropriate or unnecessary, and this contributes to the development of antimicrobial resistance (AMR) [2].

Sepsis and bacterial respiratory tract infections in particular have been linked to the generation of AMR, which carries the associated consequences of a longer duration of hospitalization, added healthcare costs and greater risk of death [3–5].

Sepsis is one of the major causes of mortality worldwide and reports of its incidence are rising [6]. According to economic studies, the financial burden for healthcare systems is considerable; in the United States (US), sepsis accounted for more than 5% of total hospital costs in 2011 ($16.7 billion US dollars [USD]) [7], and this figure is expected to be even higher in Latin America, as public health conditions are heterogeneous and there is considerable inequality in access to healthcare [8–11]. Lower respiratory tract infections (LRTIs) mainly include community-acquired pneumonia, mechanical ventilator-associated pneumonia, and acute exacerbations in patients with chronic obstructive pulmonary disease. They encompass a heterogeneous group of infections that may be either viral or bacterial in nature, the latter requiring timely antibiotic treatment.

Antibiotic stewardship programs (ASPs) have been defined as coordinated interventions designed to measure and improve appropriate use of antimicrobial treatment by promoting optimal antibiotic selection and dosing, route of administration, and treatment duration [12]. The optimization of antibiotic treatment is particularly important in intensive care units (ICUs) in view of their high rate of antibiotic use and their frequent need to treat infections caused by resistant organisms [13]. In Argentina, the human resources dedicated to ASPs are currently limited [14].

Within this context, biomarkers could be a valuable tool to indirectly support the functioning of ASPs, given their role in infection detection and assessment of disease course, as well as providing guidance for appropriate antibiotic use. Procalcitonin (PCT) is a candidate biomarker that helps distinguish between bacterial and non-bacterial infections and can predict therapeutic responses to antimicrobial treatments [15, 16]. PCT is a peptide precursor of the hormone calcitonin, which is responsible for controlling serum calcium concentrations. In inflammatory conditions such as sepsis, PCT is synthesized by virtually all of the cells in the body [17, 18]. PCT can be detected in the blood 3–4 hours after a bacterial stimulus and inflammatory response, reaching its highest concentration after 14–25 hours, and has a half-life of 22–35 hours after the bacterial stimulus has been treated. Consequently, PCT is considered a valuable prognostic biomarker that assists with predictions of clinical outcome in patients with sepsis and LRTIs [19–21]. A number of studies have demonstrated the utility of using PCT-guided algorithms in reducing antibiotic use in patients both with sepsis and LRTIs [22–24].

Health economic evaluations play a fundamental role in health policy-related decision-making [25]. An economic analysis, conducted in the US, reports the impact of PCT use on payers’ budgets when guiding antibiotic dosing [26]. To our knowledge, there are no local analyses available to date for low- and middle-income countries. Therefore, the aim of the present study was to estimate the yearly healthcare and budget impact on the Argentinian health system after the incorporation of a PCT-algorithm for guiding antibiotic prescription and dosing in hospitalized patients with suspected sepsis or LRTI.

## Methods

**Fig 1:**
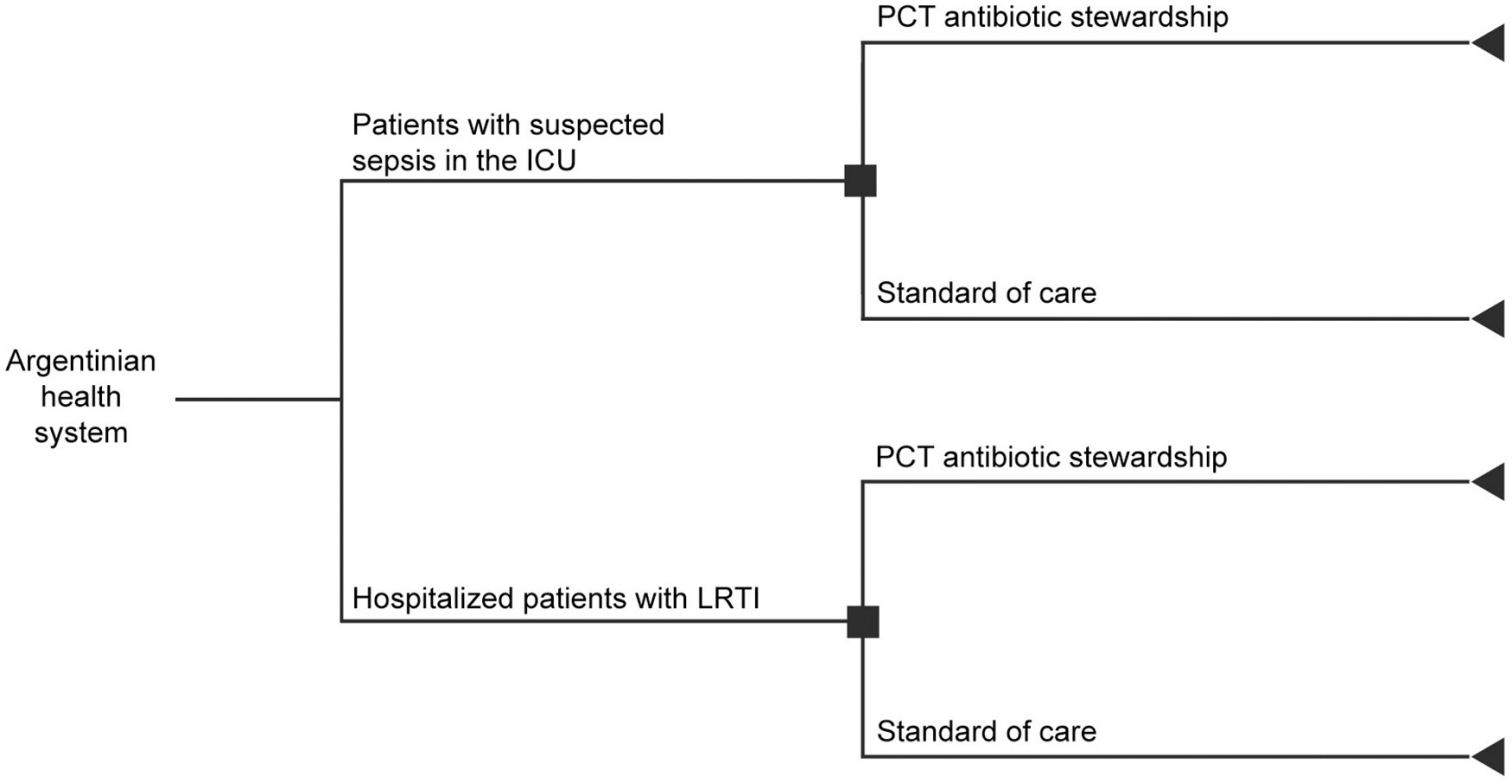
Model structure. Decision tree adapted for Argentina. ICU, intensive care unit; LRTI, lower respiratory tract infection; PCT, procalcitonin. Adapted from Mewes JC, Pulia MS, Mansour MK, Broyles MR, Nguyen HB, Steuten LM. The cost impact of PCT-guided antibiotic stewardship versus usual care for hospitalized patients with suspected sepsis or lower respiratory tract infections in the US: a health economic model analysis. PLOS One. 2019; 14(4):e0214222 [26].

The budget and healthcare impact were estimated using an adapted version of a previously published decision tree model [26] (Fig 1), which simulates the cost and clinical outcomes per individual for each of two treatment arms: intervention versus comparator. The intervention scenario comprised adding the use of PCT algorithms to routine practice to initiate and/or discontinue antibiotic treatments, and the comparator was the standard of care in Argentina, where PCT algorithms are not routinely used.

The analysis was conducted from the perspective of the aggregate Argentinian health system. The population included all patients with suspected sepsis in the ICU and hospitalized patients with LRTI (assumed to be community-acquired pneumonia or mechanical ventilator-associated pneumonia).

The model focused on cost and outcomes differences between intervention and standard of care. As the duration of the analysis was 1 year, neither inflation nor discounted rates were utilized. The main study outcomes were the budget impact per year on the Argentinean health system (USD) and the budget impact per year per person (USD) for both the intervention and standard of care. Secondary outcomes included the length of antibiotic treatment and the number of antibiotic resistant and *Clostridioides difficile (C*. *Difficile)* cases expected per year. The study followed the Consolidated Health Economic Evaluation Reporting Standards (CHEERS) statement for the reporting of results of economic assessments and the International Society for Pharmacoeconomics and Outcomes (ISPOR) budget impact analysis principles of good practice [27, 28].

The model parameters were initially obtained from a highly sensitive bibliographic search. Two reviewers independently performed a non-systematic search of web-based databases (MEDLINE, The Cochrane Central Register of Controlled Trials, EMBASE and LILACS), which included articles written in English, Spanish and Portuguese. The search results were submitted to a panel of local experts for validation, using a Delphi-like method. The panel included three critical care specialists, two pulmonologists, and three infectious diseases experts. The validation process was carried out virtually in stages. A questionnaire was designed containing the results of the bibliographic search, which each expert validated individually and anonymously based on their own scientific judgement. The anonymous responses of all participants were shared, and a new round of individual responses to the original questionnaire was carried out. Finally, a group meeting was held to verbally discuss pending disagreements, include additional considerations, and to reach a consensus.

### Model parameters

Table 1 shows the parameters used to perform all analyses. It includes the base case values (to obtain results for the deterministic base case), the minimum and maximum values (used for sensitivity analyses), and the respective data sources. S1 Table shows extended model parameters and probability distribution assumptions for sepsis and LRTI. The methods to estimate each of the inputs are described below.

**Table 1:** Model parameters for sepsis and LRTI. Epidemiological, resource use, effectiveness of PCT antibiotic stewardship and unit costs. Argentina, 2020.

Parameter	Base	Min	Max	Sources
**Sepsis patients in ICU**				
**Epidemiology**				
Cases per year, N	131,100	50,000	212,200	[29]
Hosp *C*. *diff* infections, %	2.5	1.9	3.1	[32]
AMR in general population, %	27.1	20.0	35.0	[31]
**Resource use**				
**SoC**				
Days on AB therapy	10.0	7.0	21.0	Consensus (based on [10])
Add LoS on gen ward for AMR, days	4.6	3.5	5.8	[26]
Add LoS on gen ward for *C*. *diff*, days	8.5	6.4	10.6	[26]
**PCT**				
N of PCT tests, per patient	5.0	3.0	6.5	Consensus (based on [33])
**PCT AB STW effectiveness, %**				
Reduction of days on AB therapy	15.8	24.1	7.5	[34]
Reduction of Hosp *C*. *diff*	60.0	48.0	72.0	[32]
Reduction of AMR in gen pop, per % unit reduction of days on AB therapy	3.2	2.4	4.0	See methods
**LRTI patients**				
**Epidemiology**				
Cases per year, N	216,919	204,885	229,570	[30]
Hosp *C*. *diff* infections, %	2.5	1.9	3.1	[32]
AMR in gen population, %	20.0	15.0	27.1	Consensus (based on [31])
**Resource use**				
**SoC**				
AB prescription, %	87.7	65.8	100.0	[21]
Days on AB therapy	8.1	5.0	14.0	[26]
Patients admitted to ICU, %	14.0	10.5	17.5	[30]
Add LoS on gen ward for AMR, days	8.1	6.08	10.13	[26]
Add LoS on gen ward for *C*. *diff*, days	8.49	6.37	10.61	[26]
**PCT**				
N of PCT tests, per patient	2.84	2.11	3.54	
**PCT AB STW effectiveness, %**				
Reduction of AB prescription	16.10	10.80	21.50	[21]
Reduction of days on AB therapy	30	33	27	[21]
Reduction of Hosp *C*. *diff*	60.00	48.0	72.0	[32]
Reduction of AMR in gen pop, per % unit reduction of days on AB therapy	3.20	2.40	4.00	[26]
**Unit costs, USD ($)**				
PCT test	45	40	49	[36, 37]
AB costs per day (sepsis)	180	137	224	[38–40]
AB costs per day (LRTI)	115	87	143	[38–40]
Hosp stay, gen ward, per day	164	123	205	[41]
Hosp stay, ICU, per day	345	258	431	[41]
Hosp stay in isolation, per day	281	211	352	[41]

AB, antibiotic; AB STW, antibiotic stewardship; Add, additional; AMR, antimicrobial resistance; *C*. *diff*, *Clostridioides difficile*; gen, general; Hosp, hospital; ICU, intensive care unit; LoS, length of stay; LRTI, lower respiratory tract infection; N, number; PCT, procalcitonin; pop, population; SoC, standard of care; USD, US dollars.

#### Epidemiology

As there is lack of epidemiological data available for Argentina, the annual cases of sepsis in Argentina were calculated from the projected incidence rate of 290 per 100 thousand estimated by Machado et al in Brazil [29]. The range of variability was determined according to the expert panel suggestions. For LRTI patients, given the lack of local estimates, data from the study by Lopardo et al [30] for community-acquired pneumonia were used, adjusted for the percentage of hospitalized cases (68%). The percentage of antibiotic resistance was estimated as an average of the resistance reported in Argentina for the main microorganisms that cause sepsis and LRTI [31]. The incidence of *C*. *difficile* infections was obtained from Broyles [32], as no local estimates were available.

#### Resource use

For sepsis patients, the length of antibiotic treatment was estimated according to expert opinion, based on the analysis from Estenssoro [10], a multi-center study including 49 ICUs in Argentina. The panel agreed to consider the mean antibiotic treatment in 10.0 [range: 7.0;21.0] days to better represent the current situation. For hospitalized LRTI patients, it was estimated that 87.7% [range: 65%;100%] would be prescribed antibiotics [21], for a length of 8.1 [range: 5.0;14.0] days [26]. Based on Lopardo 2018 [30], it was estimated that 14% of LRTI patients would be admitted to the ICU. The additional length of stay in a general ward due to AMR was 4.6 [range: 3.5;5.8] days for sepsis and 8.1 [range: 6.1;10.1] days for LRTIs; for *C*. *difficile* infections it was 8.5 days [range: 6.4;10.6] for both sepsis and LRTI (obtained from Mewes [26]). The mean PCT determinations per patient for the intervention were estimated as 5.0 [range: 3.0;6.5] and 2.8 [range: 2.1;3.5] for patients with sepsis and LRTI, respectively, according to Schuetz [33] and expert opinion.

#### Effectiveness of PCT antibiotic stewardship

The reduction in the length of antibiotic treatment due to PCT was estimated as 15.8% [range: 24.1%;7.5%] and 30% [range: 33%;27%] for sepsis and LRTI, respectively, according to Andriolo [34] and Schuetz [21]. Regarding reduction in length of stay in the general ward and the ICU expected from the use of PCT, the results of the bibliography analysis were not conclusive [22, 34, 35]. For this reason, and according to the expert panel opinion, we decided not to include a reduction for this parameter. The expected reduction of hospital *C*. *difficile* infection rate was taken from Broyles [32] (60% [range: 72%;48%]). To estimate the reduction in AMR we followed the same methods as in Mewes [26], which consisted of multiplying a 3.2% reduction in AMR rate by the reduction in the antibiotic therapy length.

#### Unit costs estimates

The cost of each PCT test was estimated by multiplying the number of biochemical units suggested for PCT (n = 75) by the Unified Confederation of Biochemicals in Argentina (CUBRA) [36] and the average of biochemical units values agreed for 2020 between the main healthcare payers in Argentina and Confederación Bioquímica de Córdoba [37] times $0.6 [range: $0.5;$0.7] USD. The average costs of antibiotic treatment per day for sepsis and LRTI were calculated as a weighted average between the percentage of use for each of the most regularly prescribed drugs and its dose in Argentina, and its expected daily costs (S2–S4 Tables). The percentage of use was estimated with a survey to the expert panel participants and consensus was reached through an iterative process. The costs of antibiotics were initially obtained from public databases, such as Kairos [38] and alfaBeta [39]. For each antibiotic, we calculated the average price for all available drugs. Considering these are retail prices, we assumed them to be the maximum values of the range. The minimum range values were approximated using a 39% discount from the retail price, based on estimations from the Argentinian Ministry of Economics [40]. The base case values were approximated as an average between the maximum and minimum values. This approach was validated informally with a tender specialist in Argentina. For hospital stays, we used the database of costs from the Institute of Clinical Effectiveness and Health Policy (IECS) which provides estimates for each healthcare sub-sector [41]. Weighted averages were calculated considering the distribution of patients per sub-sector of the health system [42]. Costs are expressed in USD considering an exchange rate of 80 Argentine peso per 1 USD as of November 2020 [43].

### Sensitivity analysis

The impact of parameter uncertainty on results was analyzed with deterministic and probabilistic sensitivity analyses. The deterministic sensitivity analysis that allowed the identification of the drivers of results by means of univariate variations across the ranges of parameters (Table 1) is summarized in a tornado plot. The probabilistic sensitivity analysis is a joint assessment of the impact of the parameter-associated uncertainty and consisted of simulating the result 1,000 times using Monte Carlo simulations, assuming that the variation across the ranges of parameters had been distributed according to specific probability distributions. The latter were selected according to technical recommendations [44], with the distribution parameters estimated from the mean values and standard deviations using the method of moments. The mean values were calculated as the base values of the parameters and the standard deviations were estimated as the difference between the maximum and minimum values divided by four. This simplified approach to calculating the standard deviation assumed that the minimum and maximum values represent the upper and lower 95% CIs within a normal distribution. A dependency between variables was considered for the number of PCT tests per person and the length of the antibiotic therapy, i.e. the higher or lower the number of PCT tests per person, the higher or lower the length of antibiotic therapy.

## Results

Based on the assumptions made by the model, adding the use of PCT algorithms to routine practice to administer antibiotic treatment to patients with suspected sepsis in the ICU and hospitalized LRTI patients in Argentina, the health system could avoid, per year: 734.5 [95% CI: 1,105.2;438.8] thousand antibiotic treatment days, 7.9 [95% CI: 18.5;8.5] thousand antibiotic-resistant cases, and 5.1 [95% CI: 6.7;4.2] thousand *C*. *difficile* cases. By doing so, the health system would save $422.4 [95% CI: $935;$267] USD per patient per year, which means saving $83.0 [95% CI: $183.6;$57.7] million USD for the entire health system and $0.4 [95% CI: $0.9;$0.3] million USD for a healthcare provider with 1,000 cases per year of sepsis and LRTI patients (Table 2). Using Monte Carlo simulations, the probability of cost saving is 100% (S1 Fig). When considering the sepsis and LRTI patient groups separately, the results were similar. For LRTI, the probability of cost saving is 100% (S2 Fig), and for the sepsis group, 85% (S3 Fig).

**Table 2:** Expected results. Healthcare impact, cost categories, per patient and budget impact. Sepsis and LRTI patients. Standard of care, PCT strategy and differences. Year 1. Costs expressed in US dollars. Argentina, 2020.

	Sepsis			LRTI			Total
	SoC	PCT	Difference (95% CI)	SoC	PCT	Difference (95% CI)	Difference (95% CI)
**Healthcare impact, per year, thousands**							
AB days	1,311	1,103.6	-207.4	1,757	1,229.9	-527.1	-734.5
			(-420.1;-78.0)			(-844.9;-277.7)	(-1,105.2;-438.8)
AB resistant cases	35.6	34.9	-0.7	38	30.8	-7.3	-7.9
			(-1.1;-0.3)			(-17.9;-7.8)	(-18.5;-8.5)
*C*. *diff* cases	3.3	1.3	-2.0	4.8	1.6	-3.2	-5.1 (-6.7;-4.2)
			(-2.6;-1.4)			(-4.6;-2.4)	
Costs categories, per patient, per year, USD [$]*							
PCT tests	0	226	226.4	0	128	128.4	354.8
			(159;307)			(95;163)	(273;447)
Antibiotics	1,802	1,517	-285.2	820	481	-338.6	-623.7
			(-569;-100)			(-718;-204)	(-1,118;-398)
AMR (due to ΔHosp LoS)	351	345	6.6	400	324	-76.3	-82.9
			(11;3)			(-218;-74)	(-225;-79)
*C*. *diff* infection (due to ΔHosp LoS)	60	24	-35.8	52	18	-34.8	-70.7
			(-53;-22)			(-59;-22)	(-104;-49)
Total cost difference	-	-	-101.1	-	-	-321.3	-422.4
			(-345;50)			(-774;-231)	(-935;-267)
Budget impact, per year, millions of USD [$]*							
Health system	-	-	-13.3	-	-	-69.7	-83.0
			(-45.2;6.6)			(-168.0;-50.1)	(-183.6;-57.7)
Per 1,000 patients	-	-	-0.1	-	-	-0.3	-0.4
			(-0.3;0.1)			(-0.8;-0.2)	(-0.9;-0.3)
**Probability of saving budget (%)**			85			100	100

AB, antibiotic; ABS, antibiotic stewardship; AMR, antimicrobial resistance; *C*. *diff*, *Clostridioides difficile*; Hosp, hospital; ICU, intensive care unit; LRTI, lower respiratory tract infection; PCT, procalcitonin; SoC, standard of care; USD, US dollars; ΔLoS, difference in length of stay.

*No inflation, assuming a market share of 100%.

**Fig 2:**
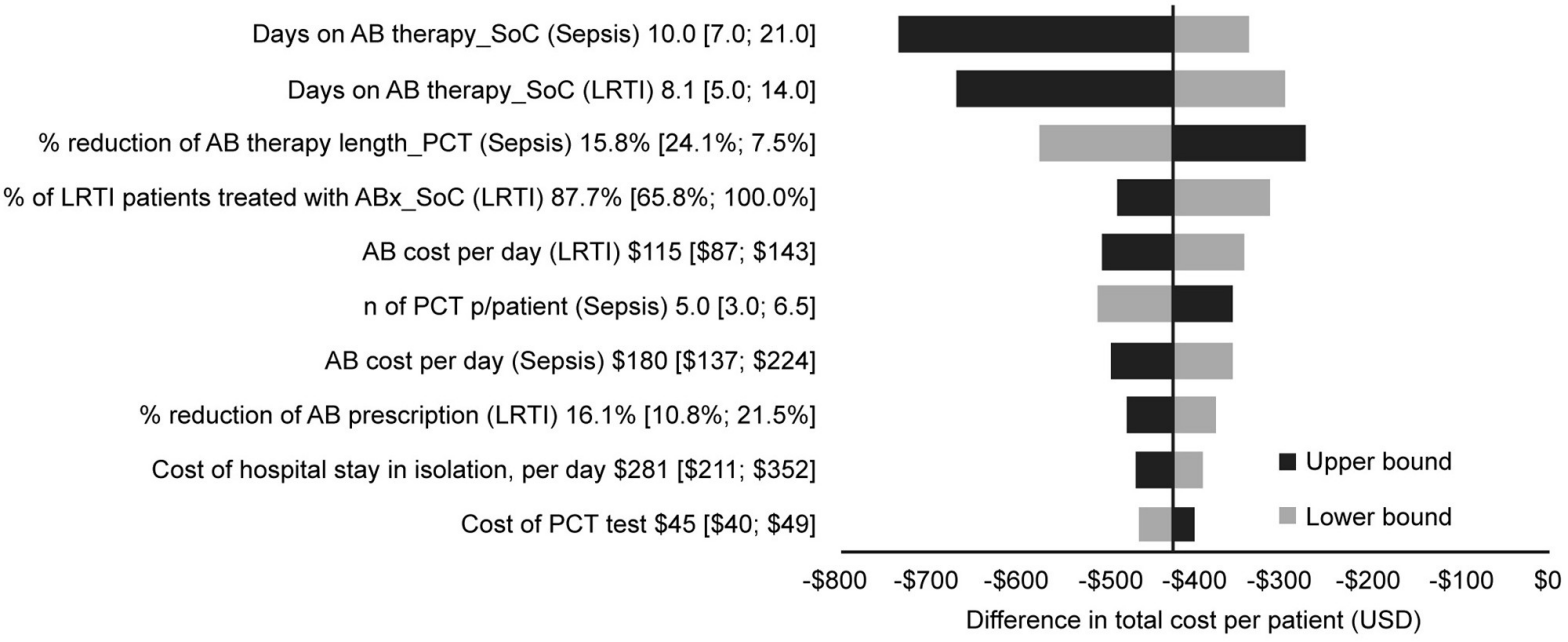
Tornado analysis. Impact of parameter variations on costs results per patient. Argentina, 2020. *The figure shows how much the base case result (−$422*.*4 USD) would change if parameters were the maximum or minimum value instead of the base case*. *For example*, *the base case value for “Days on AB therapy_SoC (Sepsis)” was 10*.*0*. *If this parameter is changed to its minimum value of 7*.*0 (instead of 10*.*0) the model predicts a result of −$337 USD (grey bar)*. *On the contrary*, *if it is changed to 21 (its maximum value) the model result would be −$736 USD*. AB, antibiotic; ABx, antibiotics; LRTI, lower respiratory tract infection; PCT, procalcitonin; SoC, standard of care; USD, US dollars.

**Fig 3:**
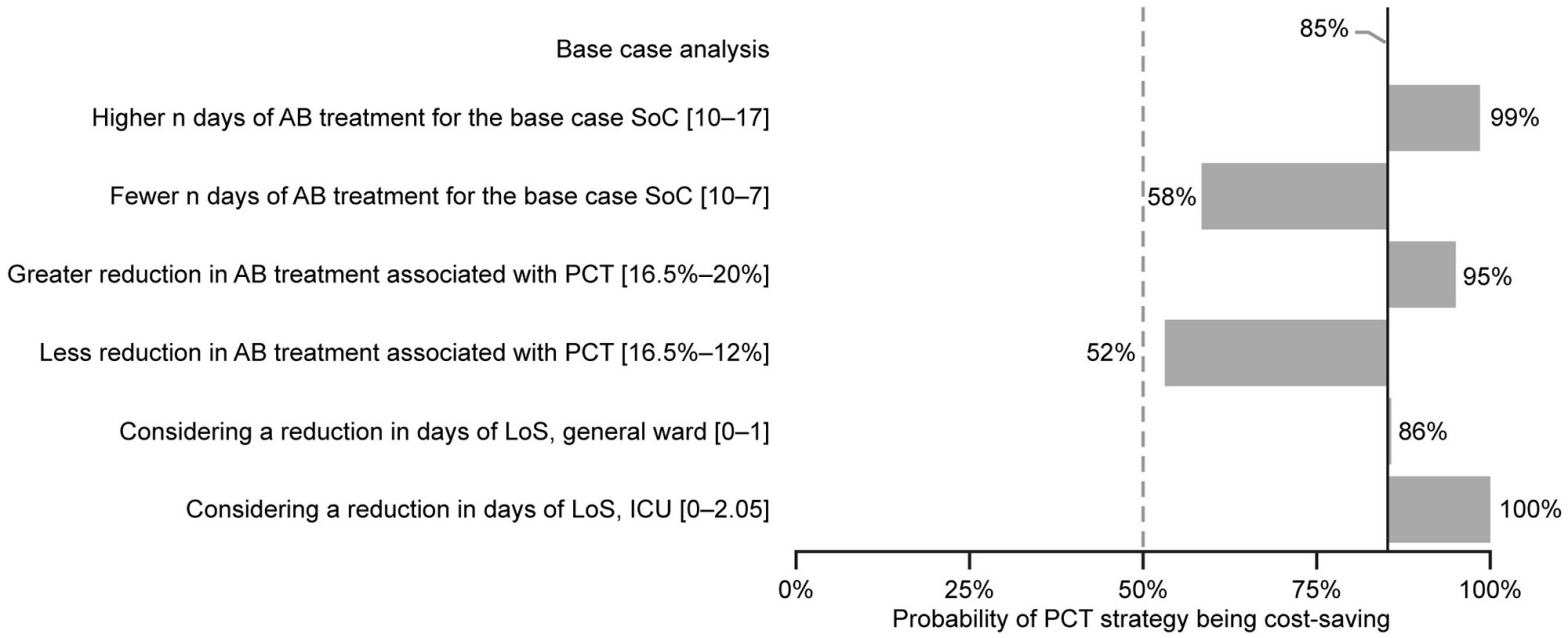
Scenario analysis. Probability of saving costs for PCT strategy according to different scenarios. Argentina, 2020. AB, antibiotic; ICU, intensive care unit; LoS, length of stay; n, number; PCT, procalcitonin; SoC, standard of care.

The sensitivity analysis (Fig 2) for the aggregate population suggests that the main variables of the models are: the length of antibiotic treatment for the standard of care; the expected length and antibiotic use reduction due to the intervention; the costs of antibiotic treatment per day; and the number of PCT tests per patient for sepsis patients. As presented in the scenario analysis in Fig 3, any variable, with its associated range of variation, that reaches the dotted threshold line (>50%, more probable to have savings than additional costs; ˂50%, more probable to have additional costs than savings), meaning that there is parameter uncertainty according to the assumptions in Table 1, is not strong enough to change the aggregate base case results. When analyzing the sepsis and LRTI patient groups separately, similar results were observed (S4 and S5 Figs), but the percentage reduction in antibiotic therapy length related to PCT use for sepsis may reach the dotted threshold line ($0 USD), meaning that if there was a reduction in magnitude (e.g., 7.5% instead of 15.8%) for this sub-group, the model resulted in additional costs instead of savings. S4 Fig shows that 15% of cases cross to the positive budget impact zone, resulting in a probability of 85% savings.

## Discussion

AMR is currently a major challenge globally, especially for low- and middle-income countries [45]. Studies have shown that up to 50% of antibiotic prescriptions in hospitals are either inappropriate or unnecessary, and this contributes to the development of antimicrobial resistance [2]. In 2015, the World Health Organization released a global action plan on AMR, and ASPs were one of its cornerstones [46]. In this context, PCT biomarkers emerge as a promising tool to support clinical decisions.

Healthcare systems in every country need to manage their resources and plan their actions wisely, with efficiency as a fundamental value; studies such as health economic evaluations are key in supporting and informing decision-making. This budget impact analysis sought to understand the financial implications associated with the incorporation of PCT biomarkers into routine practice in Argentina and to help Argentinian policy makers evaluate the potential benefits and resource implications. To our knowledge, it is the first analysis of this type conducted in Argentina.

Our results suggest that by implementing PCT antibiotic treatment-guided strategies, a considerable benefit in health outcomes can be obtained through reduction in the number of cases of AMR and *C*. *difficile* infections, and therefore, decreased costs. The health system can avoid, per year: 734.5 [95% CI: 1,105.2;438.8] thousand antibiotic treatment days; 7.9 [95% CI: 18.5;8.5] thousand antibiotic-resistant cases; and 5.1 [95% CI: 6.7;4.2] thousand *C*. *difficile* cases. This would save $422.4 [95% CI: $935;$267] USD per patient per year, meaning $83.0 [95% CI: $183.6;$57.7] million USD of savings for the entire health system and $0.4 [95% CI: $0.9;$0.3] million USD for a healthcare provider with 1,000 cases per year of sepsis and LRTI patients. The key factor driving these results is a reduction in antibiotic therapy, in terms of both the length of the therapy and the number of patients receiving therapy. Our results show savings for LRTI patients to be three times higher than for sepsis patients. This difference comes from a higher reduction in antibiotic treatment, i.e. shortened treatments and fewer patients receiving antibiotic therapy, and an expected lower number of PCT tests per patient.

Although we found results to be robust on an aggregate level, with a 100% probability of cost savings, caution must be used when focusing only on sepsis patients in the ICU. In the sepsis patient subgroup, results favor the strategy based on PCT but are less robust compared with the LRTI subgroup. In this case, the probability of cost savings drops to 85%, and in the worst-case scenario it is reduced to 52%. Nevertheless, it is important to highlight that these results were obtained while maintaining a conservative point of view, especially by considering a high cost for PCT testing and not accounting for potential benefits associated with the reduction of the length of hospital stay, particularly in the ICU. Other studies, such as Mewes et al [26], have included ICU-related costs that largely benefit the intervention with PCT; however, the evidence around a reduction in the length of stay in the ICU is not robust [22, 34, 35]. Moreover, it is also relevant to mention that these results are more applicable to institutions without shortened treatment guidelines as savings are reduced if the duration of antimicrobial treatment is shortened.

One limitation of this study is that local literature on the use of PCT-guided therapy is very limited and, therefore, many of the parameters used in the model were derived from international literature. To counterbalance this limitation, we sought guidance from a panel of local experts to discuss the implications of using international evidence and provide insights of the Argentinian healthcare system to better represent the local setting. However, the reliance on expert opinion to generate insights, combined with the low number of experts who were consulted, is a limitation itself, therefore, we ensured that we appropriately assessed the uncertainty around opinion-derived input. Additionally, the epidemiological data and the modalities of antibiotic use are supported by both regional and local multicentre studies. Another limitation of the study is the high uncertainty associated with cost parameters in Argentina due to a high inflation index and the permanent variation of the USD currency exchange rate. Efforts were made to include this uncertainty in the various sensitivity analyses, but results could change from the base case if the main parameters are not captured between the ranges considered. The results of this study suggest that there are cost-saving benefits with implementing PCT-guided strategies versus the standard of care; however, given the implications of implementing this paradigm shift, future studies should include administrative costs such as training and infrastructure as part of the model.

## Conclusion

AMR and *C*. *difficile* infections can be reduced in Argentina with considerable cost savings by implementing PCT-guided strategies versus the standard of care. Although we found results to be robust on an aggregate level, caution must be used when focusing only on sepsis patients in the ICU.

## Supporting information

S1 Fig Scatterplot. Antibiotic-resistant (ABR) cases avoided and additional costs in US dollars (USD). Sepsis and lower respiratory tract infection patients. Argentina, 2020.
(TIF)

S2 Fig Scatterplot. Antibiotic-resistant (ABR) cases avoided and additional costs in US dollars (USD). Lower respiratory tract infection patients. Argentina, 2020.
(TIF)

S3 Fig Scatterplot. Antibiotic-resistant (ABR) cases avoided and additional costs in US dollars (USD). Sepsis patients. Argentina, 2020.
(TIF)

S4 Fig Tornado analysis. Impact of univariate variations on the difference in total costs per patient. Sepsis patients. Argentina, 2020. AB, antibiotic; ABx, antibiotics; *C*. *difficile*, *Clostridioides difficile;* hosp, hospital; LoS, length of stay; PCT, procalcitonin; SoC, standard of care; USD, US dollars. The dotted line represents the threshold for the probability of additional costs vs. savings.
(TIF)

S5 Fig Tornado analysis. Impact of univariate variations on the difference in total costs per patient. LRTI patients. Argentina, 2020. AB, antibiotic; ABR, antibiotic resistant; ABx, antibiotics; LoS, length of stay; LRTI, lower respiratory tract infection; PCT, procalcitonin; SoC, standard of care; USD, US dollars. The dotted line represents the threshold for the probability of additional costs vs. savings.
(TIF)

S1 Table Extended model parameters and probability distribution assumptions. Epidemiological, resource use, effectiveness of PCT antibiotic stewardship and unit costs. Argentina, 2020. AB, antibiotic; AB STW, antibiotic stewardship; Add, additional; AMR, antimicrobial resistance; C. diff, Clostridioides difficile; gen, general; Hosp, hospital; ICU, intensive care unit; LoS, length of stay; LRTI, lower respiratory tract infection; N, number; PCT, procalcitonin; pop, population; SoC, standard of care; USD, US dollars.
(DOCX)

S2 Table Daily antibiotic cost estimation for hospitalized LRTI patients. Antibiotic therapies, weighted average and cost per day in US dollars. Upper bound of parameter range. Argentina. October 2020. h, hours; IV, intravenous; LRTI, lower respiratory tract infection. *Weighted average estimated by the expert panel’s opinions. See S3 Table for more information about unit costs.
(DOCX)

S3 Table Daily antibiotic cost estimation for sepsis patients. Antibiotic therapies, weighted average and cost per day in US dollars. Upper bound of parameter range. Argentina. October 2020. h, hours. *Weighted average estimated by the expert panel’s opinions. See S3 Table for more information about unit costs.
(DOCX)

S4 Table Daily antibiotic cost treatment estimation. Cost per day in US dollars. Argentina. October 2020. h, hours; IM, intramuscular; IV, intravenous. **Sources:** https://ar.kairosweb.com/ and https://www.alfabeta.net/precio/srv (October 2020).
(DOCX)
