# Supplementary figures and images for: Budget impact analysis of using procalcitonin to optimize antimicrobial treatment for patients with suspected sepsis in the intensive care unit and hospitalized lower respiratory tract infections in Argentina
Source: PLoS One. 2021 Apr 30;16(4):e0250711. doi: 10.1371/journal.pone.0250711 (PMC8087000; doi:10.1371/journal.pone.0250711)

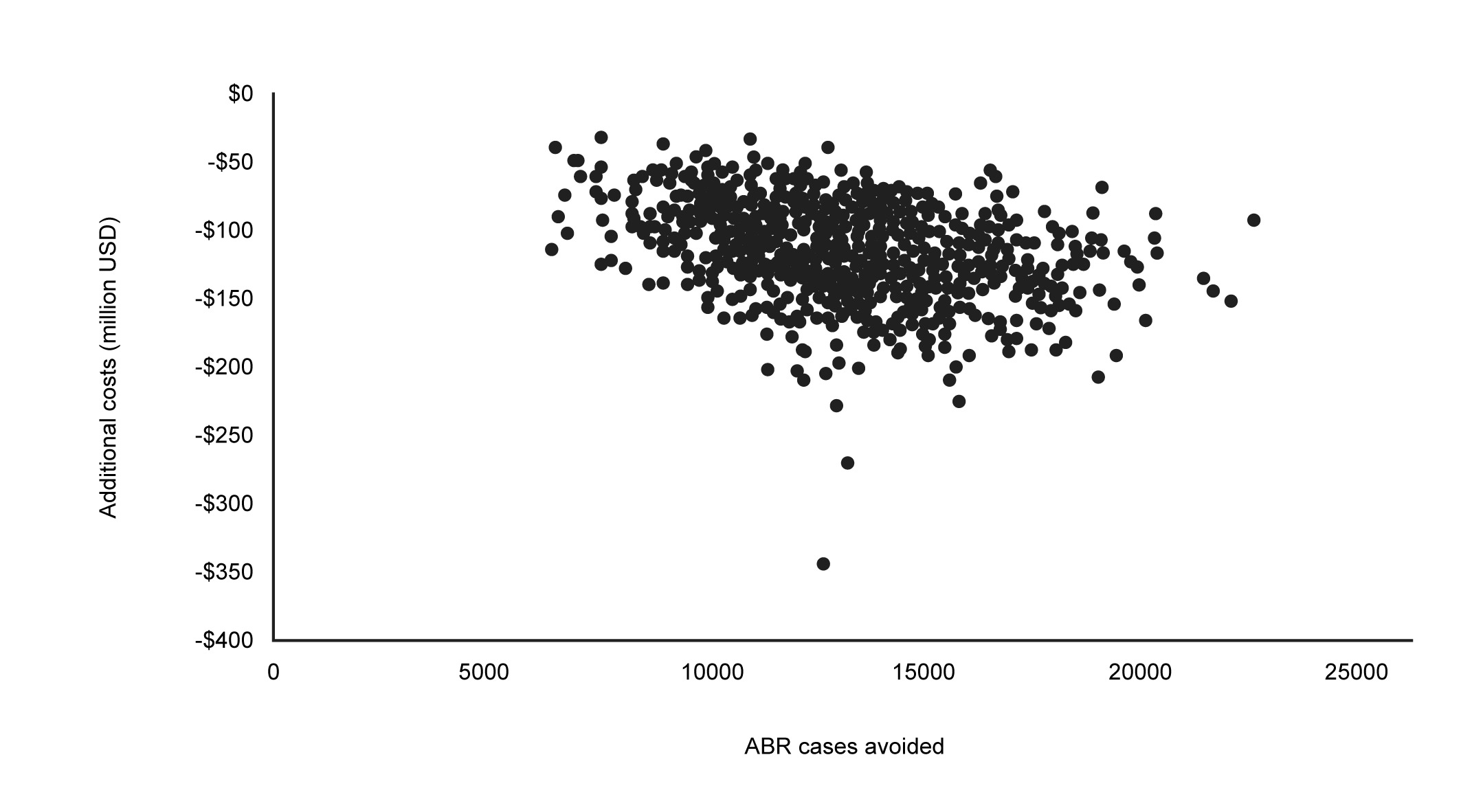

Supplement: S1 Fig — Antibiotic-resistant (ABR) cases avoided and additional costs in US dollars (USD). Sepsis and lower respiratory tract infection patients. Argentina, 2020. (TIF) [file pone.0250711.s001.tif]

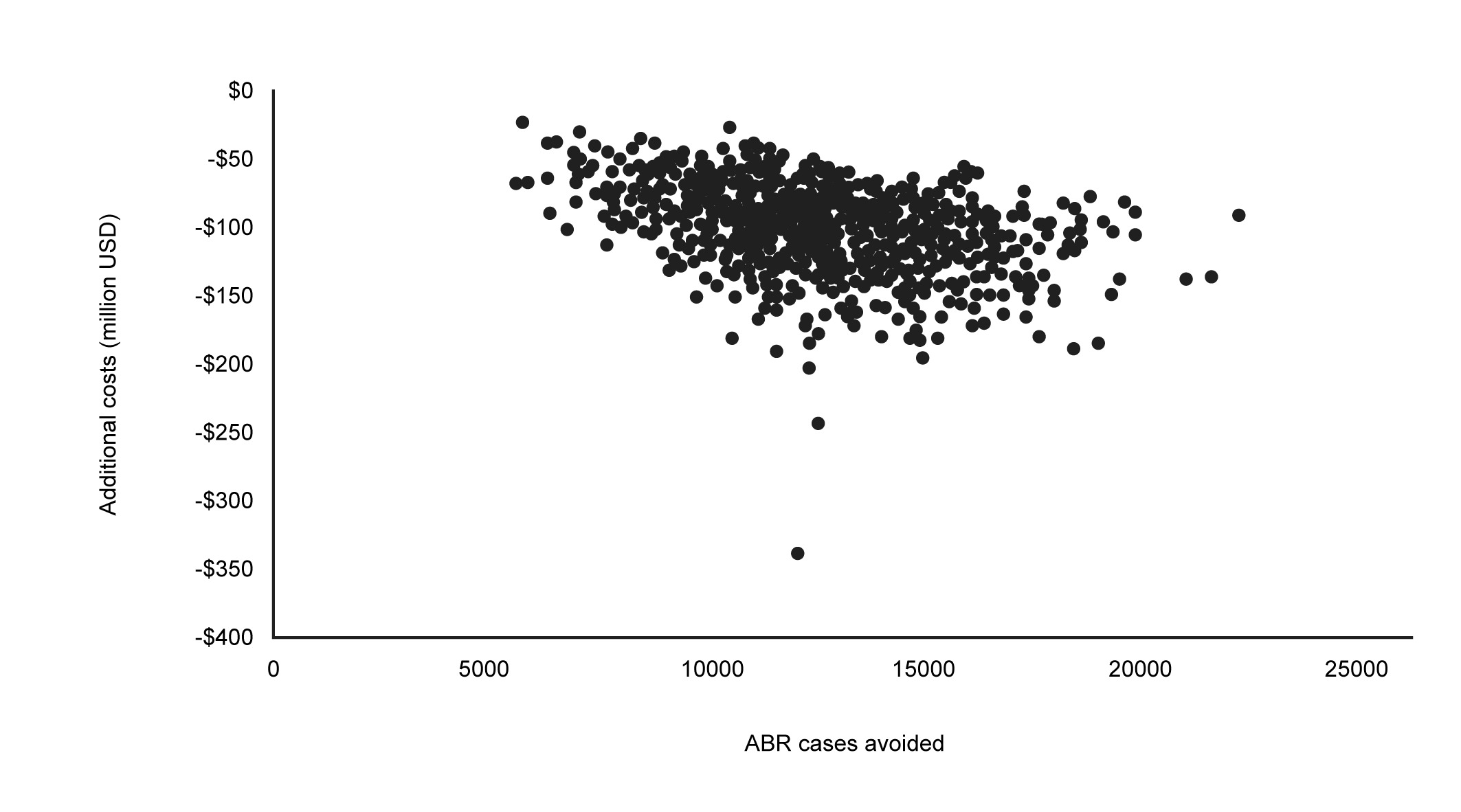

Supplement: S2 Fig — Antibiotic-resistant (ABR) cases avoided and additional costs in US dollars (USD). Lower respiratory tract infection patients. Argentina, 2020. (TIF) [file pone.0250711.s002.tif]

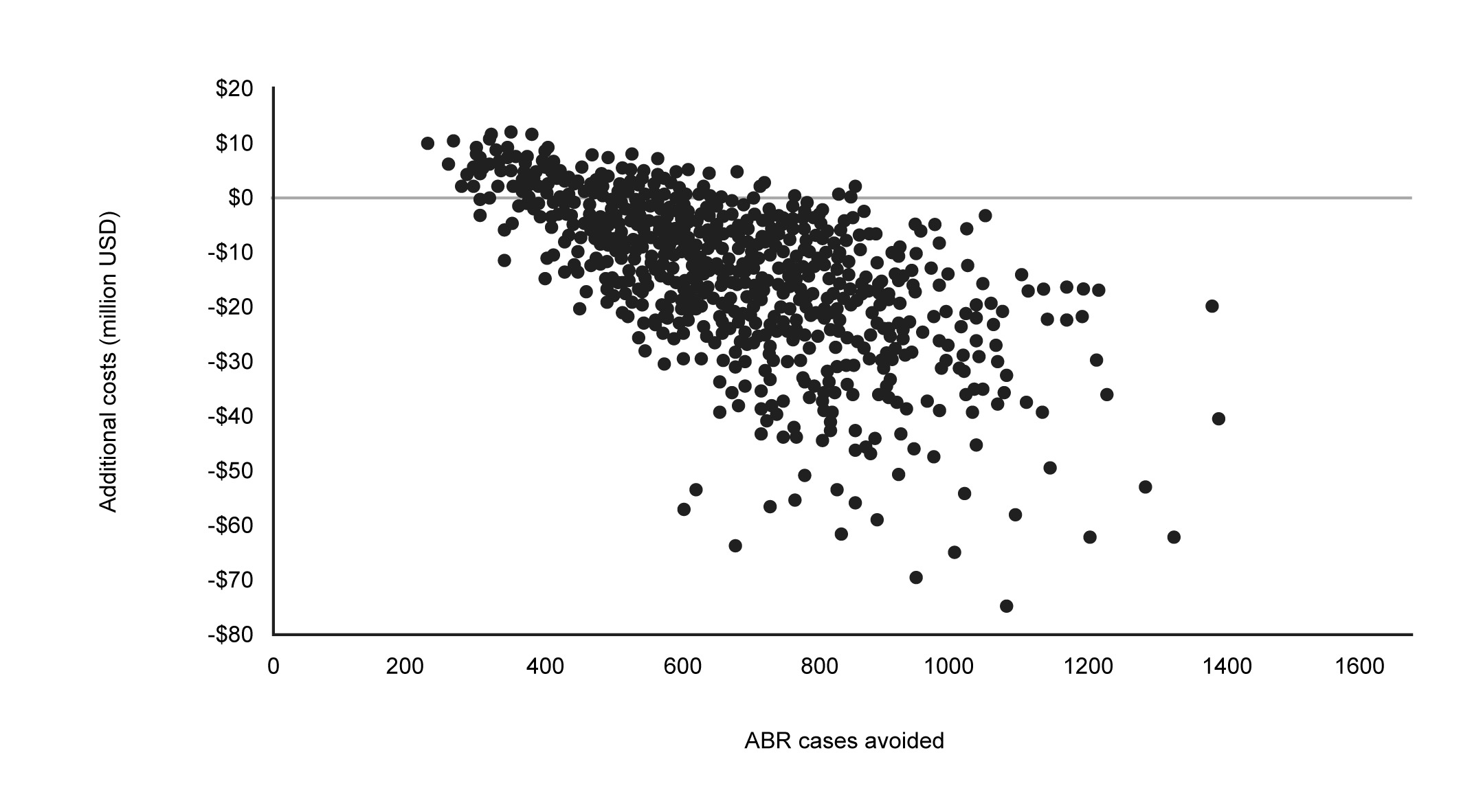

Supplement: S3 Fig — Antibiotic-resistant (ABR) cases avoided and additional costs in US dollars (USD). Sepsis patients. Argentina, 2020. (TIF) [file pone.0250711.s003.tif]

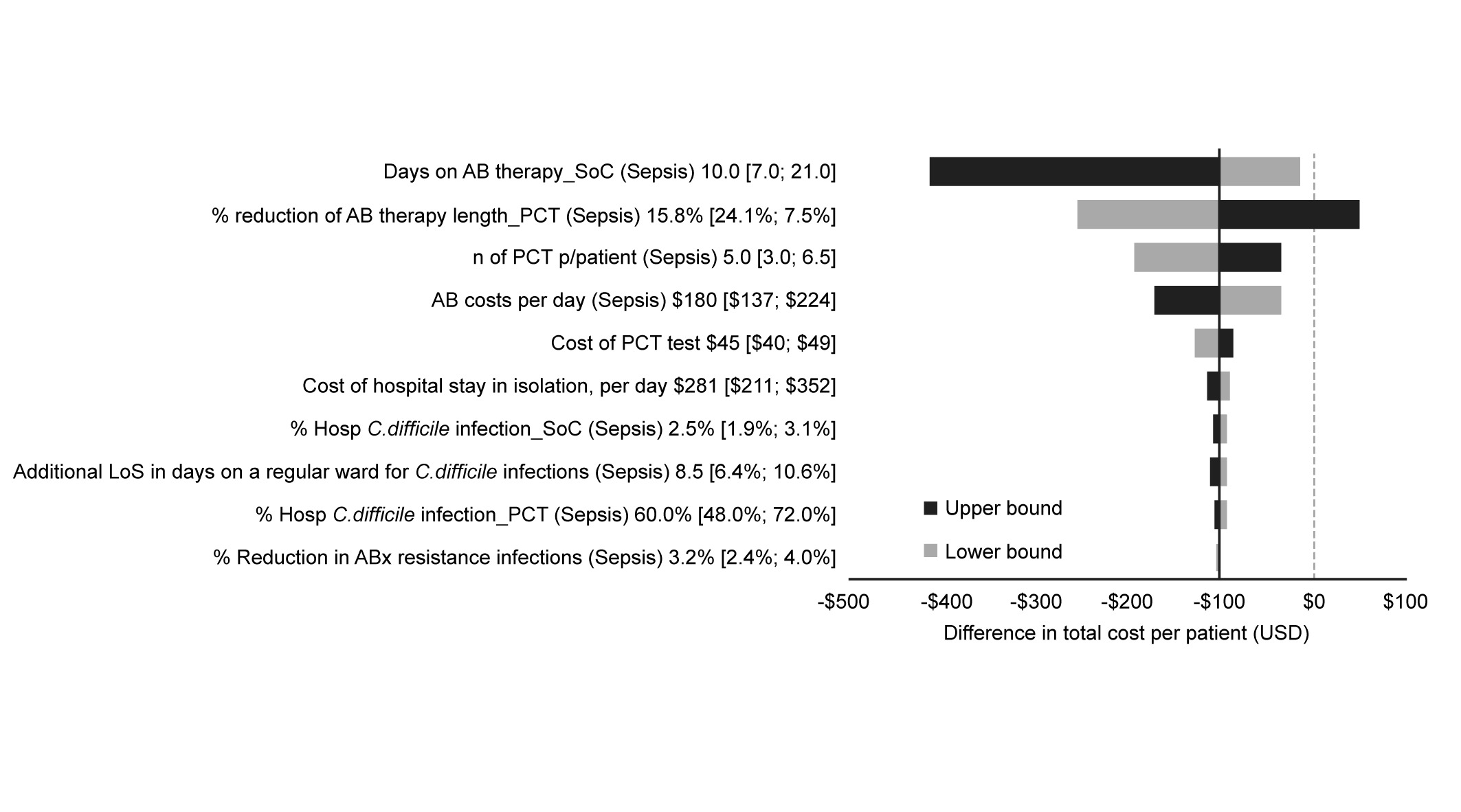

Supplement: S4 Fig — Impact of univariate variations on the difference in total costs per patient. Sepsis patients. Argentina, 2020. AB, antibiotic; ABx, antibiotics; C. difficile, Clostridioides difficile; hosp, hospital; LoS, length of stay; PCT, procalcitonin; SoC, standard of care; USD, US dollars. The dotted line represents the threshold for the probability of additional costs vs. savings. (TIF) [file pone.0250711.s004.tif]

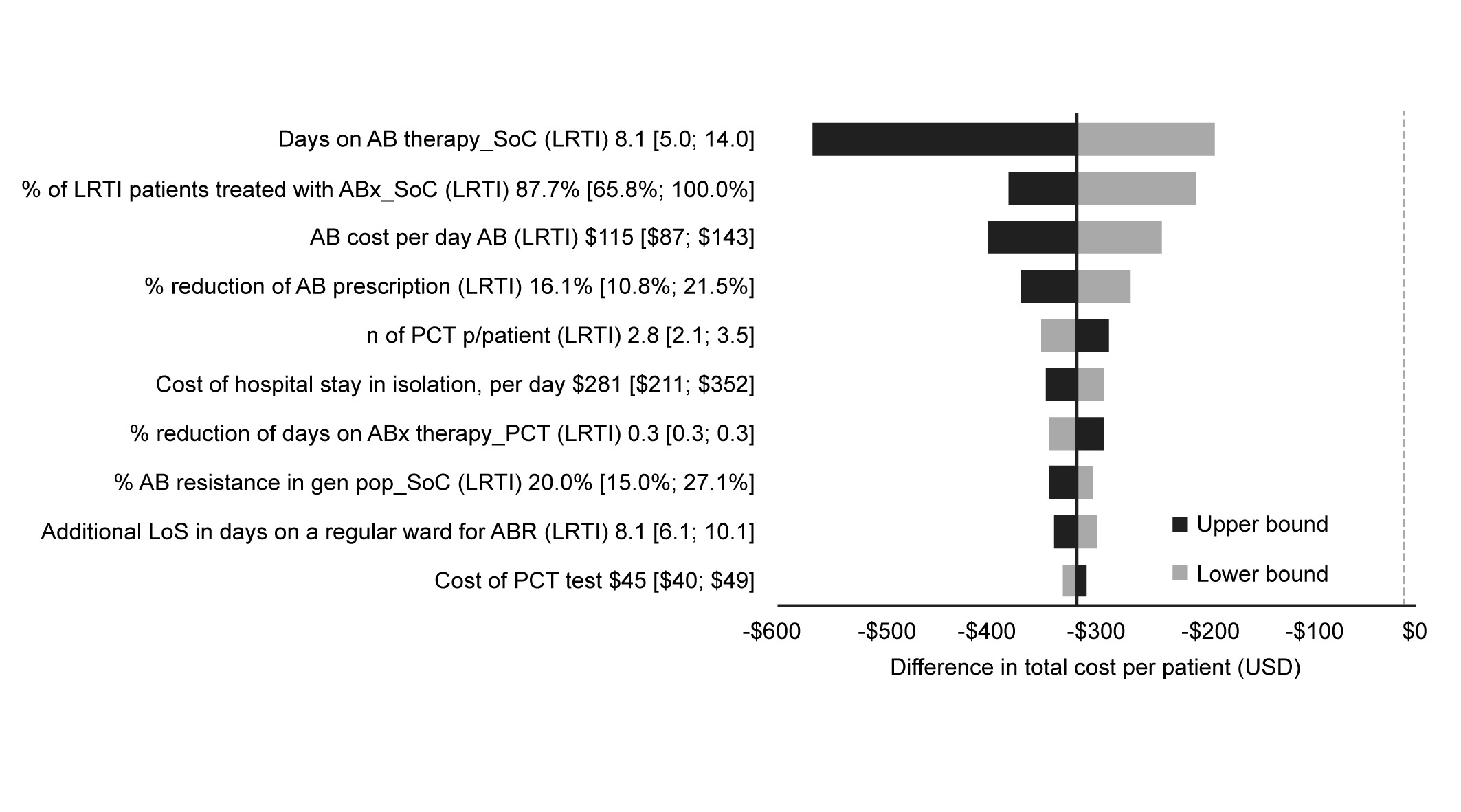

Supplement: S5 Fig — Impact of univariate variations on the difference in total costs per patient. LRTI patients. Argentina, 2020. AB, antibiotic; ABR, antibiotic resistant; ABx, antibiotics; LoS, length of stay; LRTI, lower respiratory tract infection; PCT, procalcitonin; SoC, standard of care; USD, US dollars. The dotted line represents the threshold for the probability of additional costs vs. savings. (TIF) [file pone.0250711.s005.tif]
