# Supplementary material for: Budget impact analysis of using procalcitonin to optimize antimicrobial treatment for patients with suspected sepsis in the intensive care unit and hospitalized lower respiratory tract infections in Argentina
Source: PLoS One. 2021 Apr 30;16(4):e0250711. doi: 10.1371/journal.pone.0250711 (PMC8087000; doi:10.1371/journal.pone.0250711)
Supplement: S1 Table — Epidemiological, resource use, effectiveness of PCT antibiotic stewardship and unit costs. Argentina, 2020. AB, antibiotic; AB STW, antibiotic stewardship; Add, additional; AMR, antimicrobial resistance; C. diff, Clostridioides difficile; gen, general; Hosp, hospital; ICU, intensive care unit; LoS, length of stay; LRTI, lower respiratory tract infection; N, number; PCT, procalcitonin; pop, population; SoC, standard of care; USD, US dollars. (DOCX) [file pone.0250711.s006.docx]

**S1 Table. Extended model parameters and probability distribution assumptions. Epidemiological, resource use, effectiveness of PCT antibiotic stewardship and unit costs. Argentina, 2020.**

| **Parameter** | | | | **Base** | **Min** | **Max** | **Probability distribution and parameters** | **Sources** |
| --- | --- | --- | --- | --- | --- | --- | --- | --- |
| **Sepsis patients in ICU** | | | |  |  |  |  |  |
|  | **Epidemiology** | | |  |  |  |  |  |
|  |  |  | Cases per year, N | 131,100 | 50,000 | 212,200 | Gamma  (10.5; 12,542.35) | [[29]](https://www.zotero.org/google-docs/?13IvgV) |
|  |  |  | Hosp *C. diff* infections, % | 2.5 | 1.9 | 3.1 | Beta  (62.4; 2,432.63) | [[32]](https://www.zotero.org/google-docs/?oxZWfF) |
|  |  |  | AMR in general population, % | 27.1 | 20.0 | 35.0 | Beta  (37.9; 101.73) | [[31]](https://www.zotero.org/google-docs/?rBkzCL) |
|  | **Resource use** | | |  |  |  |  |  |
|  |  | **SoC** | |  |  |  |  |  |
|  |  |  | Days on AB therapy | 10.0 | 7.0 | 21.0 | Gamma  (8.2; 1.23) | Consensus (based on [[10]](https://www.zotero.org/google-docs/?YzTUbp)) |
|  |  |  | Add LoS on gen ward for AMR, days | 4.6 | 3.5 | 5.8 | Gamma  (64.0; 0.07) | [[26]](https://www.zotero.org/google-docs/?iawgYN) |
|  |  |  | Add LoS on gen ward for *C. diff*, days | 8.5 | 6.4 | 10.6 | Gamma  (64.0; 0.13) | [[26]](https://www.zotero.org/google-docs/?H7wDc0) |
|  |  | **PCT** | |  |  |  |  |  |
|  |  |  | N of PCT tests, per patient | 5.0 | 3.0 | 6.5 | Gamma  (32.7; 0.15) | Consensus (based on [[33]](https://www.zotero.org/google-docs/?7Nvxvk)) |
|  | **PCT AB STW effectiveness, %** | | |  |  |  |  |  |
|  |  |  | Reduction of days on AB therapy | 15.8 | 24.1 | 7.5 | Beta (12.1; 64.54) | [[34]](https://www.zotero.org/google-docs/?5nlCIP) |
|  |  |  | Reduction of Hosp *C. diff* | 60.0 | 48.0 | 72.0 | Beta (39.4; 26.27) | [[32]](https://www.zotero.org/google-docs/?qPsIHR) |
|  |  |  | Reduction of AMR in gen pop, per % unit reduction of days on AB therapy | 3.2 | 2.4 | 4.0 | Beta (61.9; 1,873.08) | See methods |
| **LRTI patients** | | | |  |  |  |  |  |
|  | **Epidemiology** | | |  |  |  |  |  |
|  |  |  | Cases per year, N | 216,919 | 204,885 | 229,570 | Gamma  (1,235.5; 175.57) | [[30]](https://www.zotero.org/google-docs/?zqwttQ) |
|  |  |  | Hosp *C. diff* infections, % | 2.5 | 1.9 | 3.1 | Beta  (62.4; 2,432.63) | [[32]](https://www.zotero.org/google-docs/?WjhdO3) |
|  |  |  | AMR in gen population, % | 20.0 | 15.0 | 27.1 | Beta (34.5; 138.10) | Consensus (based on [[31]](https://www.zotero.org/google-docs/?GVUid2)) |
|  | **Resource use** | | |  |  |  |  |  |
|  |  | **SoC** | |  |  |  |  |  |
|  |  |  | AB prescription, % | 87.7 | 65.8 | 100.0 | Beta (12.0; 1.69) | [[21]](https://www.zotero.org/google-docs/?V977Uq) |
|  |  |  | Days on AB therapy | 8.1 | 5.0 | 14.0 | Gamma  (13.0; 0.63) | [[26]](https://www.zotero.org/google-docs/?9YExGY) |
|  |  |  | Patients admitted to ICU, % | 14.0 | 10.5 | 17.5 | Beta  (54.9; 337.24) | [[30]](https://www.zotero.org/google-docs/?x4o5Gj) |
|  |  |  | Add LoS on gen ward for AMR, days | 8.1 | 6.08 | 10.13 | Gamma  (64.0; 0.13) | [[26]](https://www.zotero.org/google-docs/?u04pFO) |
|  |  |  | Add LoS on gen ward for *C. diff*, days | 8.49 | 6.37 | 10.61 | Gamma  (64.0; 0.13) | [[26]](https://www.zotero.org/google-docs/?d9laS4) |
|  |  | **PCT** | |  |  |  |  |  |
|  |  |  | N of PCT tests, per patient | 2.84 | 2.11 | 3.54 | Gamma  (62.8; 0.05) |  |
|  | **PCT AB STW effectiveness, %** | | |  |  |  |  |  |
|  |  |  | Reduction of AB prescription | 16.10 | 10.80 | 21.50 | Beta  (30.5; 158.30) | [[21]](https://www.zotero.org/google-docs/?TZQO6o) |
|  |  |  | Reduction of days on AB therapy | 30 | 33 | 27 | Beta  (210.6; 491.38) | [[21]](https://www.zotero.org/google-docs/?BrdOWk) |
|  |  |  | Reduction of Hosp *C. diff* | 60.00 | 48.0 | 72.0 | Beta (39.4; 26.27) | [[32]](https://www.zotero.org/google-docs/?SyTL4O) |
|  |  |  | Reduction of AMR in gen pop, per % unit reduction of days on AB therapy | 3.20 | 2.40 | 4.00 | Beta  (61.9; 1,873.08) | [[26]](https://www.zotero.org/google-docs/?poCxGV) |
| **Unit costs, USD ($)** | | | |  |  |  |  |  |
|  |  |  | PCT test | 45 | 40 | 49 | Gamma  (450.9; 0.10) | [[36](https://www.zotero.org/google-docs/?ic2J2u),[37]](https://www.zotero.org/google-docs/?BfreiH) |
|  |  |  | AB costs per day, sepsis | 180 | 137 | 224 | Gamma  (68.2; 2.64) | [[38–40]](https://www.zotero.org/google-docs/?Iw0V7j) |
|  |  |  | AB costs per day, LRTI | 115 | 87 | 143 | Gamma  (68.2; 1.69) | [[38–40]](https://www.zotero.org/google-docs/?tmekC6) |
|  |  |  | Hosp stay, gen ward, per day | 164 | 123 | 205 | Gamma  (64.0; 2.56) | [[41]](https://www.zotero.org/google-docs/?z8hIfZ) |
|  |  |  | Hosp stay, ICU, per day | 345 | 258 | 431 | Gamma  (64.0; 5.38) | [[41]](https://www.zotero.org/google-docs/?JiLMyN) |
|  |  |  | Hosp stay in isolation, per day | 281 | 211 | 352 | Gamma  (64.0; 4.40) | [[41]](https://www.zotero.org/google-docs/?8TkRTa) |

AB, antibiotic; AB STW, antibiotic stewardship; Add, additional; AMR, antimicrobial resistance; C. diff, Clostridioides difficile; gen, general; Hosp, hospital; ICU, intensive care unit; LoS, length of stay; LRTI, lower respiratory tract infection; N, number; PCT, procalcitonin; pop, population; SoC, standard of care; USD, US dollars.
