# Supplementary material for: Budget impact analysis of using procalcitonin to optimize antimicrobial treatment for patients with suspected sepsis in the intensive care unit and hospitalized lower respiratory tract infections in Argentina
Source: PLoS One. 2021 Apr 30;16(4):e0250711. doi: 10.1371/journal.pone.0250711 (PMC8087000; doi:10.1371/journal.pone.0250711)
Supplement: S2 Table — Antibiotic therapies, weighted average and cost per day in US dollars. Upper bound of parameter range. Argentina. October 2020. h, hours; IV, intravenous; LRTI, lower respiratory tract infection. *Weighted average estimated by the expert panel’s opinions. See S3 Table for more information about unit costs. (DOCX) [file pone.0250711.s007.docx]

**S2 Table. Daily antibiotic cost estimation for hospitalized LRTI patients. Antibiotic therapies, weighted average and cost per day in US dollars. Upper bound of parameter range. Argentina. October 2020.**

| **Antibiotic for hospitalized LRTI** | | **Weighted average* (%)** | **Cost per day (USD [$])** |
| --- | --- | --- | --- |
|  | Ampicillin-Sulbactam (1.5 g IV every 6 h) + Clarithromycin (500 mg IV every 12 h) | 34 | 45.80 |
|  | Meropenem (1 g every 8 h) + Colistin (150 mg every 12 h) | 19 | 340.70 |
|  | Meropenem (1 g every 8 h) + Amikacin (15 mg/kg every 24 h) | 5 | 303.20 |
|  | Ceftazidime (2 g every 8 h) + Colistin (150 mg every 8 h) + Amikacin (15 mg/kg every 24 h) | 2 | 133.50 |
|  | Ceftriaxone (2 g each 24 h) | 9 | 18.80 |
|  | Piperacillin/Tazobactam (4.5 g every 6 h) +/- Amikacin (15 mg/kg every 24 h) | 14 | 211.60 |
|  | Colistin (150 mg every 12 h) + Amikacin (15 mg/kg every 24 h) | 5 | 60.80 |
|  | Colistin (150 mg every 12 h) + Tigecycline (50 mg every 12 h) | 2 | 103.70 |
|  | Colistin (150 mg every 12 h) + Tigecycline (50 mg every 12 h) + Fosfomycin (4 g every 6 h) | 5 | 190.80 |
|  | Vancomycin (1g every 12 h) | 3 | 55.80 |
|  | Others | 4 | - |
|  | **Total (upper bound of interval)** | **100** | **143.40** |

h, hours; IV, intravenous; LRTI, lower respiratory tract infection. *****Weighted average estimated by the expert panel’s opinions. See Table S3 for more information about unit costs.
