# Supplementary material for: Budget impact analysis of using procalcitonin to optimize antimicrobial treatment for patients with suspected sepsis in the intensive care unit and hospitalized lower respiratory tract infections in Argentina
Source: PLoS One. 2021 Apr 30;16(4):e0250711. doi: 10.1371/journal.pone.0250711 (PMC8087000; doi:10.1371/journal.pone.0250711)
Supplement: S4 Table — Cost per day in US dollars. Argentina. October 2020. h, hours; IM, intramuscular; IV, intravenous. Sources: https://ar.kairosweb.com/ and https://www.alfabeta.net/precio/srv (October 2020). (DOCX) [file pone.0250711.s009.docx]

**S4 Table. Daily antibiotic cost treatment estimation. Cost per day in US dollars. Argentina. October 2020.**

| **Drug** | **Concentration** | **Price (USD [$])** | **Suggested dose** | **Cost per day (USD [$])** |
| --- | --- | --- | --- | --- |
| Ceftriaxone | 1,000 mg IM injection Blister vial | 9.4 | 1 g every 12 h | 18.8 |
| Amikacin | 500 mg | 10.2 | 15 mg/kg every 24 h | 21.5 |
| Meropenem | 1 g Blister vial | 93.9 | 2 g every 8 h | 563.5 |
| Vancomycin | 500 mg Blister vial | 13.9 | 1 g every 12 h | 55.8 |
| Piperacillin/ Tazobactam | 4.5 g Blister vial | 47.5 | 4.5 g every 6 h | 190.2 |
| Colistin | 2 ml injection Blister vial + 100 mg solvent | 13.1 | 150 mg every 12 h | 39.3 |
| Imipenem | 500 mg IV Blister vial | 52.1 | 500 mg every 6 h | 208.4 |
| Tigecycline | 50 mg injection Blister vial x 10 | 321.8 | 50 mg every 12 h | 64.4 |
| Ampicillin-Sulbactam | 1.5 g injection Blister vial | 4.5 | 1.5 g IV every 6 h | 17.9 |
| Clarithromycin | 500 mg lyophilized injection | 14.0 | 500 mg IV every 12 h | 27.9 |
| Meropenem | 1 g injection Blister vial x 1 | 93.9 | 1 g every 8 h | 281.7 |
| Ceftazidime | 1,000 mg Blister vial x 100 | 8.8 | 2 g every 8 h | 53.0 |
| Colistin | 2 ml injection Blister vial + 100 mg solvent | 13.1 | 150 mg every 8 h | 59.0 |
| Ceftriaxone | 1,000 mg IM injection Blister vial x 1 | 9.4 | 2 g every 24 h | 18.8 |
| Fosfomycin | 1 g IV injection Blister vial | 5.4 | 4 g every 6 h | 87.1 |

h, hours; IM, intramuscular; IV, intravenous.

**Sources:** https://ar.kairosweb.com/ and https://www.alfabeta.net/precio/srv (October 2020)
